# Lessons Learned from Sudan Ebola Virus Disease (SUDV) Preparedness in Rwanda: A Comprehensive Review and Way Forward

**DOI:** 10.1007/s44197-023-00133-0

**Published:** 2023-06-28

**Authors:** Edson Rwagasore, Olivier Nsekuye, Ziad El-Khatib, Adeline Kabeja, Valois H. Mucunguzi, Pacifique Nizeyimana, Edward Ruseesa, Laurent Ruyange, Isabelle B. Teta, Sandrine Uwamahoro, Solange Twahirwa, Denyse Mugawaneza, Leandre Ishema, Hugor Shema, Alfred Rutagengwa, Valens Ndagijimana, Ines Itanga, Alexis Kapiteni, Jean Luc Benimana, Jean Claude Niyoyita, Bruce Rwagitinywa, Claude Mambo Muvunyi

**Affiliations:** 1 Public Health Surveillance, Emergency Preparedness and Response Division (PHS&EPR), Rwanda Biomedical Center (RBC), P.O. Box 7162, Kigali, Rwanda; 2 Department of Global Public Health, Karolinska Institutet, 17177 Solna, Sweden; 3 Bill and Joyce Cumming Institute of Global Health, University of Global Health Equity, 6955 Kigali, Rwanda; 4 World Health Organization Rwanda Country Office, P.O. Box 1324, Kigali, Rwanda

**Keywords:** Rwanda, Sudan Ebola virus disease, Preparedness, Lessons learned

## Abstract

**Background:**

Ebola Virus Disease (EVD) is a severe and often fatal illness that affects humans and has significant public health implications, including high mortality rates, strain on healthcare systems, and social and economic disruption. On 20 September 2022, Uganda declared an Ebola disease outbreak caused by the Sudan ebolavirus species. The neighboring countries of Uganda were classified by World Health Organization (WHO) as being at high risk of Sudan Ebola Virus Disease (SUDV) importation. The country of Rwanda implemented different sustainable strategies and activities to prepare and ensure a timely and effective response to SUDV outbreaks once it has arrived in the country. We aimed to highlight the sustainable strategies and activities implemented for SUDV preparedness and the subsequent lessons learnt in Rwanda.

**Methods:**

This paper reviewed the documentation on activities implemented for SUDV preparedness, with a focus on lessons learned from different countries. The paper analyzed the common themes and highlighted the key components of EVD preparedness in Rwanda after declaration of SUDV outbreak in Uganda.

**Results:**

The key components of SUDV preparedness include its readiness assessment in Rwanda, effective coordination, collaboration and leadership mechanisms, enhancing the early detection and surveillance system, effective risk communication and community engagement, capacity building of healthcare providers on case management and infection prevention and control (IPC), and continual preparedness. These components were essential to ensure timely and effective preparation and response to SUDV related outbreaks.

**Conclusion:**

A multi-sectoral approach involving all stakeholders was necessary to ensure timely and effective preparation and response. Continuous investment in preparedness, strengthening of health systems, and the review of preparedness components provided insights into the best practices for SUDV preparedness, which were essential to prevent future outbreaks and minimize their impact. This will inform other countries about the role of timely development of preparedness plans.

## Introduction

Ebola disease outbreak caused by the Sudan ebolavirus species was declared by the government of Uganda on 20 September 2022, when positivity for Ebola virus infection was confirmed for a 24-year-old man hospitalized with a wide range of symptoms, including high-grade fever, tonic convulsions, blood-stained vomit and diarrhea, and bleeding in the eyes [1]. This was the first Sudan ebolavirus outbreak in a decade in Uganda, and its fifth overall for this kind of Ebola, without a vaccine or therapeutic options available, preparedness activities are critical for response efforts. This outbreak was declared finished on 11 January 2023 as the last patient was released from care on 30 November [2].

There is no history of any confirmed cases of any Ebola virus in Rwanda. However, in previous years, EVD cases were detected in two neighboring countries, namely the Democratic Republic of Congo (DRC) and Uganda, which share borders with Rwanda where the daily average number of individuals crossing the border between DRC and Rwanda is approximately 20,000, while between Uganda and Rwanda, it is approximately 1500 individuals per day. On 1 August 2018, the Ministry of Health of the Democratic Republic of the Congo declared the tenth outbreak of Ebola Virus Disease in North Kivu Province [3]. As preparedness measures, Rwanda developed a National Preparedness Plan and trained health workers in early detection and response, educating communities about Ebola, vaccinating health workers in high-risk areas, equipping health facilities, and conducting simulation exercises to maintain a high level of readiness. Screening for Ebola symptoms at points of entry has been also done since the beginning of the outbreak in the Democratic Republic of the Congo, and has been reinforced since the confirmation of a case in the Congolese city of Goma. An Ebola Treatment Center has been set up in Rwanda and 23 isolation units have been prepared in hospitals in 15 priority districts [4].

As one of the neighboring countries of Uganda had recently active SUDV outbreak, Rwanda was at high risk of importing SUDV due to the exposure of large cross-border population movements (due to usual travel, trade, farming, social activities). Furthermore, Rwanda was receiving people coming from the affected districts of Uganda (Mubende, Kampala, Kassanda, Kyegegwa, Kagadi, Bunyangabu, Wakiso, Kampala, Masaka and Jinja). Considering the risk of importation of SUDV and from the lessons learnt to the coronavirus disease 2019 (COVID-19) pandemic—which demonstrated to the world that policy guidelines for response capacities, even in high income countries with assumed robust health systems, did not necessarily translate into practice [5]—it was imperative that the country put more efforts for SUDV preparedness to respond earlier and contain the virus effectively once arrived in Rwanda. A well-documented narrative for the activities done during the preparedness phase is needed to inform future response efforts. Thus, this case study highlights the sustainable strategies and activities for SUDV preparedness and lessons learnt in Rwanda.

## Methods

The synthesis of findings from this case study was conducted between January 2023 and April 2023, following the declaration of the Sudan Ebolavirus Disease (SUDV) outbreak in Uganda as concluded. Findings were synthesized from various sources by conducting the review of SUDV preparedness activities documents such as readiness assessment reports, contingency plans, simulation exercise reports, and field mission reports. We have also conducted a review of reports from daily and weekly situational awareness meetings and review of overall activities developed to improve the system’s readiness capacity. Many of the authors were also directly engaged in SUDV preparedness activities and participated in coordination meetings at district and country levels that provided insights into the key priorities activities for SUDV readiness in the country as well as documentation of preparedness activities within different pillars: leadership and coordination, surveillance at point of entries, health facility and in the community, laboratory, case management, IPC, safe dignified burial, risk communication and community engagement and operational support and logistics.

### Risk for Importation of Sudan Ebola Virus in Rwanda

According to WHO risk assessment among neighboring countries of Uganda, the country of Rwanda was classified among countries at risk based on important population movements to and from Uganda [6].

**Fig. 1:**
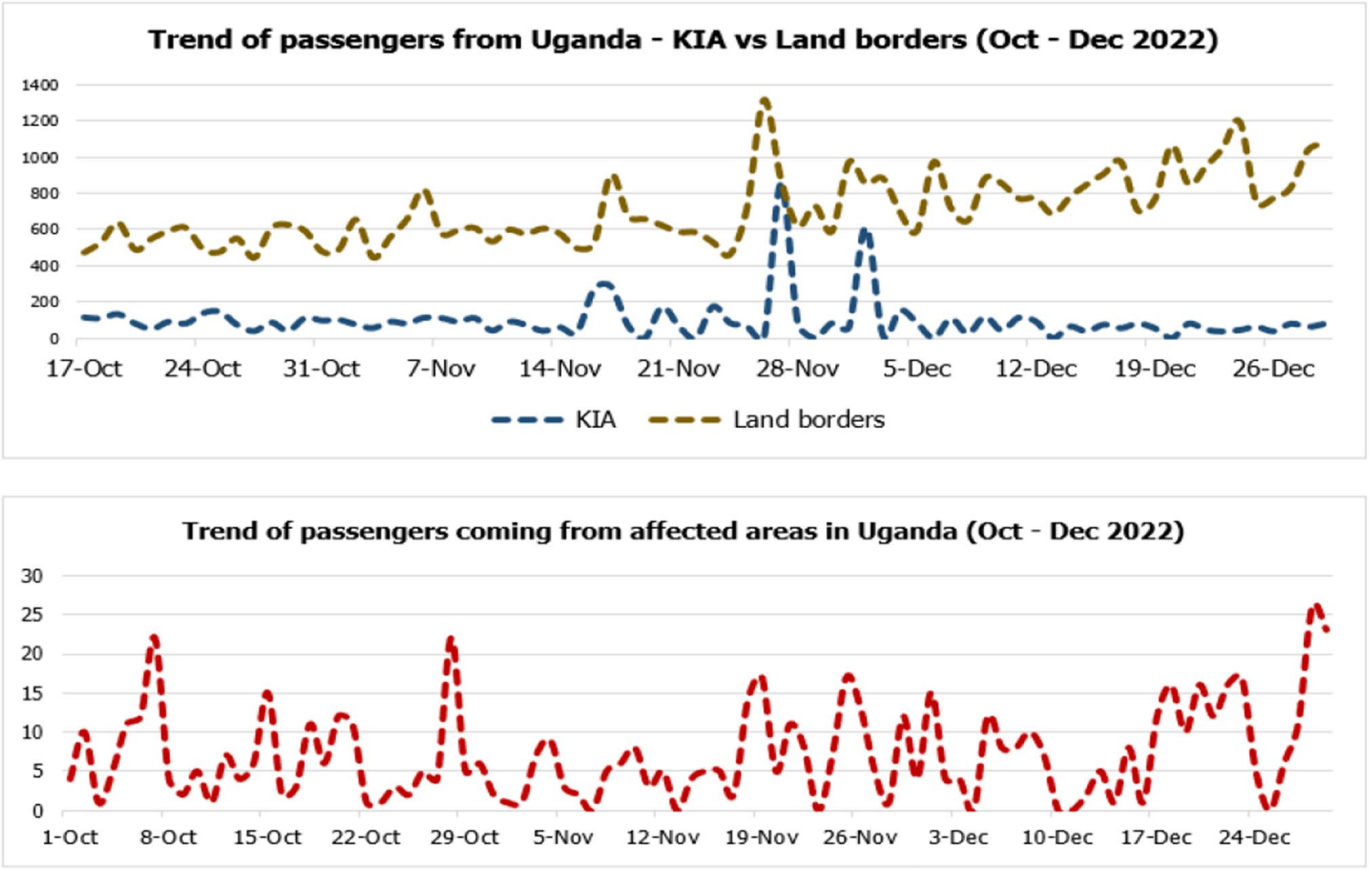
Trend of passengers from Uganda

The most likely route by which the virus could have been introduced to Rwanda is through infected people from affected areas of Uganda traveling to Rwanda. There are two options of entering Rwanda from Uganda, either through flight or using land borders. There is a direct flight connecting Rwanda and Uganda: Kigali International Airport (KIA) to/from Entebbe International Airport. There are four official land borders connecting Rwanda and Uganda, which are the Kagitumba and Buziba in the Nyagatare District, Gatuna in the Gicumbi District, and Cyanika in the Burera District. Some people might use porous borders, however, through the collaboration of local authorities and the community where everyone entering villages is notified to local leaders through the established daily reporting mechanism either using call or short messages, some people using porous borders are detected through that mechanism. On average 95 passengers were coming in Rwanda per day using flights from Uganda, while approximately 659 passengers were entering Rwanda from Uganda per day using land borders. Furthermore, seven passengers from Uganda per day on average were coming from affected areas of Uganda. See details in the Fig. 1 below.

**Fig. 2:**
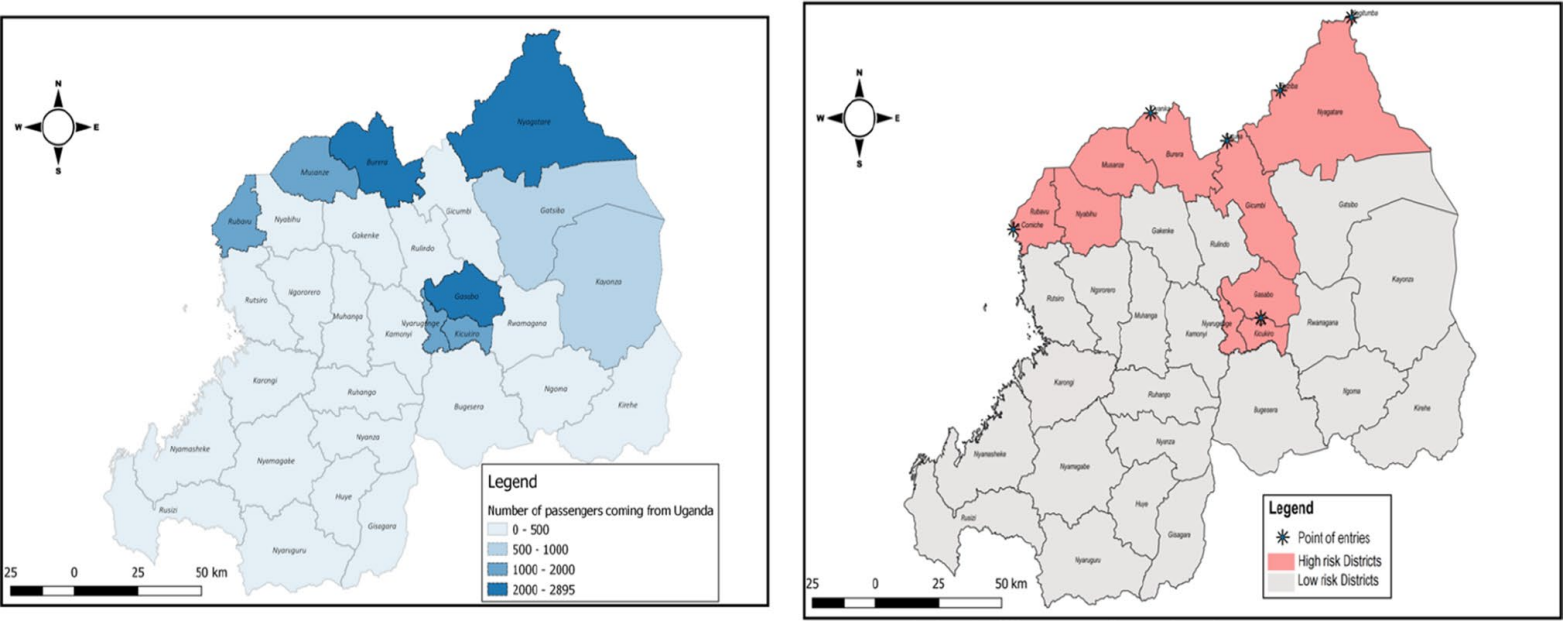
Maps of (left) Rwanda indicating the number of incoming passengers from Uganda and their destination (22 Sep -07 Oct 2022) and (right) map of Rwanda indicating the districts at high risk of SUDV

The risk assessment was conducted based on a rapid risk assessment methodology in geographical areas of Rwanda where the probability of infection was more likely to occur, and these areas were determined as priority for preparedness activities. These include districts of Rwanda bordering the country of Uganda: Nyagatare District, Gicumbi District, Burera District, Musanze District, Nyabihu District, and other districts that are highly visited by people from Uganda such as the City of Kigali and Rubavu District (Fig. 2).

### Pre-assessment for the Country Readiness

**Fig. 3:**
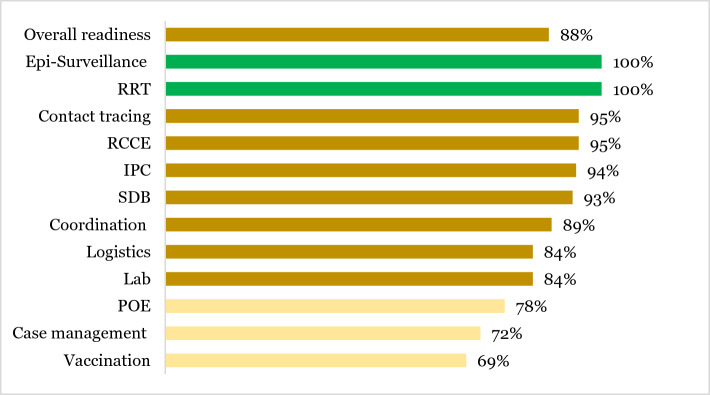
WHO checklist assessment summary* *WHO* World health organization; *RRT* rapid response team; *RCCE* risk communication and community engagement; *IPC* infection prevention and control; *SDB* safe dignified burial; *Lab* laboratory; *POE* point of entries

The pre-assessment was conducted using the Ebola Virus Disease Consolidated Preparedness Checklist adapted from WHO [7]. This assessment was conducted in September 2022 after confirmation of SUDV outbreak revealed that the country was prepared at 88% (Fig. 3).

This has been considered a very good score for the beginning. But the main gaps have been identified to guide decision makers where to prioritize in terms of SUDV preparedness. The following main gaps have been identified from different pillars as summarized in Table 1:

**Table 1:** List of the identified gaps

Pillar	Gap
Coordination	No bilateral cross-border mechanism for sharing surveillance data is established between affected and priority countries
	No tabletop/ SIMEX conducted to assess functionality of EVD Preparedness
Case management	No new therapeutics for Sudan Ebola virus
Vaccination	No guidelines and SOPs for preventive and ring vaccination strategy
	Frontliners are not vaccinated against SUDV
Point of entries (POE)	No SIMEX conducted to assess functionality of EVD preparedness at POE
	Information Education Communication (IEC) materials are not posted at all designated POE
Risk communication and community engagement (RCCE)	Key influencers (local government, politicians, journalists, community/religious leaders, security forces, etc.) were partially orientated at community level
Laboratory	Low number of PCR testing kits in store (1 kit to be used for 96 samples)
Safe dignified burial	Safe dignified burial and space not yet identified
Contact tracing	Need to improve data capturing tool for contact tracing

The gaps identified during the pre-assessment using the Ebola virus disease consolidated Preparedness checklist have been instrumental in guiding Rwanda’s strategic direction for SUDV preparedness. These identified gaps highlighted areas of vulnerability that could compromise an effective response to a SUDV outbreak. Recognizing the need for further improvement, Rwanda undertook a series of preparedness activities tailored to address these deficiencies to ensure SUDV readiness

### Coordination and Leadership

During the SUDV preparedness phase in Rwanda, coordination and leadership played a critical role. Coordination and leadership were essential for ensuring that all stakeholders worked together effectively to prevent, detect, and respond to potential EVD outbreaks.

Coordination involved bringing together all relevant stakeholders, including the Rwanda Biomedical Center, Ministry of Health, Ministry of local government, hospitals, and international partners to work together in a collaborative and efficient manner. The effective coordination enabled stakeholders to identify gaps and prioritized activities that are essential for EVD preparedness. It also ensured that resources were allocated efficiently and effectively, maximizing their impact on EVD preparedness efforts. Coordination was also critical for early detection and rapid response to potential EVD outbreaks, as it enables stakeholders to establish surveillance systems, train healthcare workers, and develop rapid response plans that can be implemented quickly in the event of an outbreak.

The following are some of the key roles that coordination and leadership played during this phase:

- Setting up a multi-sectoral coordinating committee: a multi-sectoral coordinating committee was established to oversee the preparedness efforts. This committee included representatives from different sectors such as the Ministry of Health, Ministry of local government, Security organs, and multinational partners.
- Developing and implementing a national preparedness plan and contingency plan: a national preparedness plan and contingency plan were developed and implemented. They outlined different activities to be undertaken and the roles and responsibilities of different stakeholders and provide guidance on the key actions to be taken during the preparedness phase. The contingency plan included the critical activities to be implemented within 6 months with a budget of 15,304,918 USD.
- Coordinating and conducting risk assessments: risk assessments were conducted to identify the potential sources of EVD transmission and assess the readiness of the health system to respond to an outbreak. Coordination was required to ensure that these assessments were conducted comprehensively and consistently.
- Ensuring the availability of essential resources: essential resources such as personal protective equipment, diagnostic tools, quarantine sites, and isolation centers were made available during the preparedness phase. Coordination was required to ensure that these resources were distributed or renovated appropriately and efficiently.
- Mobilizing resources: resources were mobilized from different partners and stakeholders to support the preparedness efforts. Coordination was required to ensure that these resources were mobilized effectively and efficiently.

#### Strengthening Surveillance and Early Detection

In Rwanda, viral hemorrhagic fevers (VHFs) are included in Rwanda’s Integrated Disease Surveillance and Response (IDSR) priority pathogens. As part of the surveillance system, suspect VHF cases are routinely reported by health facilities through eIDSR system. Strengthening surveillance and early detection was one of the critical components of SUDV preparedness efforts in a country to prevent the spread of the disease, reduce morbidity and mortality, and minimize the economic impact of the outbreak in the country. The following are some of the ways in which surveillance and early detection was strengthened during the preparedness phase of SUVD in Rwanda:

#### Strengthening Surveillance System at Point of Entries (POEs)

At the level of POEs, Rwanda considered screening of passengers coming from Uganda as the first step for an early detection of any suspected person for SUDV and as one of the measures to prevent the introduction of SUVD in the country. To reduce the likelihood of SUDV being introduced into the country, an algorithm for screening at POEs was developed:

All passengers entering the country were screened for temperature, and their travel history was also assessed. Passengers from Uganda presenting any EVD-like symptoms were immediately isolated in designated areas by the government and tested for SUDV. Contact tracing of all co-passengers was also planned to be initiated once tested positive.

**Fig. 4:**
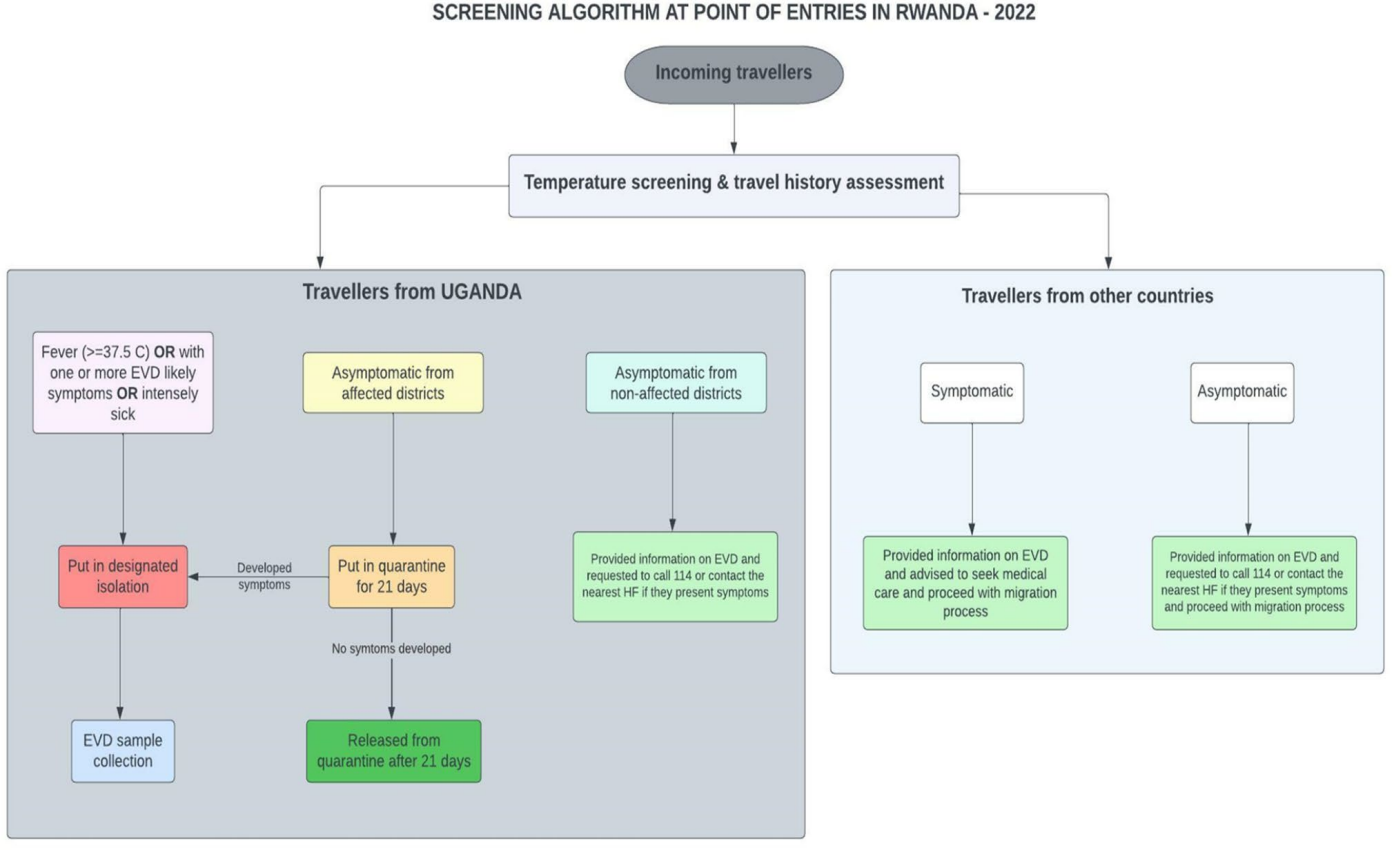
Screening algorithm for SUDV at POEs

Asymptomatic passengers coming from affected areas in Uganda were put under quarantine for 21 days in designated quarantine sites within all districts bordering Uganda. Details on the screening algorithm at all points of entries are described in Fig. 4.

#### Strengthening Surveillance System in Health Facilities

Disease surveillance among health facilities involves the collection, analysis, and reporting of data on potential disease cases in health facilities, including health centers, hospitals, and private clinics. During the SUVD preparedness phase in Rwanda, to strengthen SUDV surveillance among health facilities, there was a development and dissemination of SUDV case definition and SUDV clinical management guidelines in health facilities. These documents included criteria for SUDV case identification, screening tools, and procedures for the isolation and management of suspected cases. The national level sent these documents to all district hospitals, which in turn distributed them to all health facilities within their catchment areas. Healthcare workers from all health facilities and all private clinics from high-risk districts and some selected private clinics in the city of Kigali were also trained on how to identify potential SUDV cases, how to use screening tools effectively, and how to report suspected EVD cases to public health officials.

#### Developing and Implementing Early Warning Systems

Early warning systems can help to detect potential EVD outbreaks before they become widespread. Early warning systems involve the use of data to identify potential EVD cases and alert public health officials to the possibility of an outbreak. During the preparedness phase, the country had an opportunity to initiate and implement early warning systems that were effective, reliable, and timely.

1. Using of an innovative system for passenger’s follow-up (WELTEL)

WELTEL (Wireless Emergency Telemedicine) system is a telemedicine system that provides medical assistance to remote and underserved areas using wireless communication technology. The system was developed by the World Health Organization (WHO) in collaboration with the International Telecommunication Union (ITU) and the International Atomic Energy Agency (IAEA). During outbreaks and pandemics, it is used to monitor and support cases and contacts in the community via mobile phones [8, 9].

**Fig. 5:**
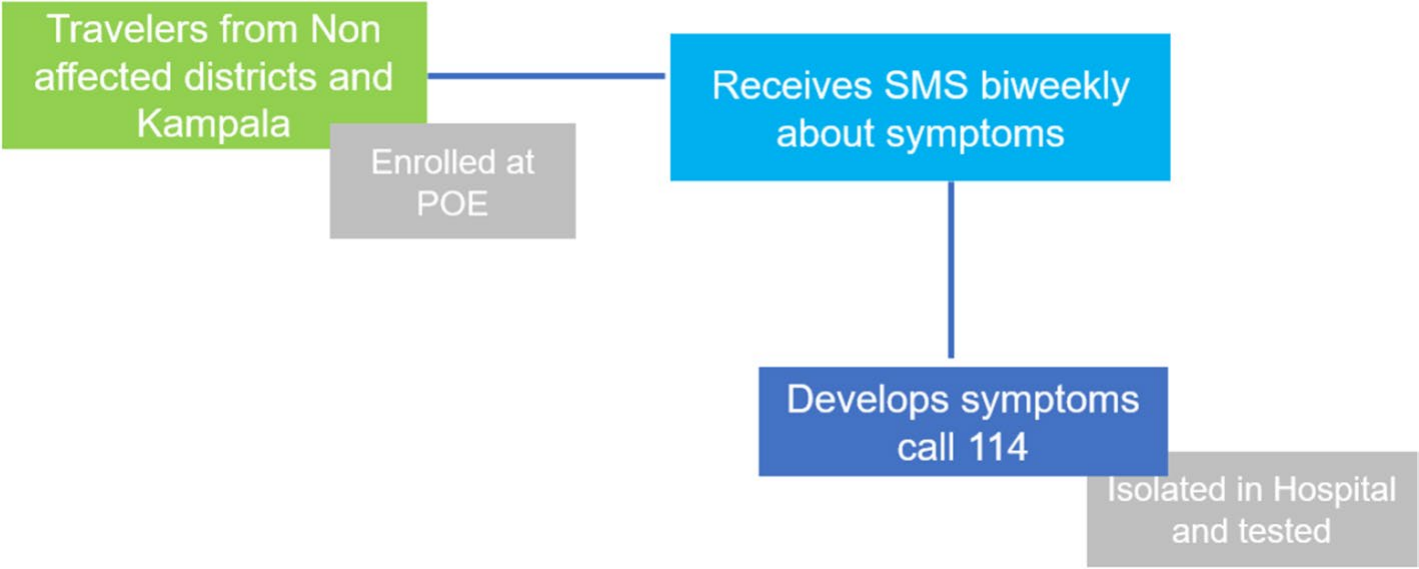
WELTEL passenger’s follow-up system flowchart

This system was used to enroll asymptomatic passengers coming from Uganda in non-affected areas in the system, and a short message was sent to them on a bi-weekly basis for 21 days after their entry into the country to follow-up on their status and determine whether SUVD symptoms developed. Around 15,000 messages have been sent to passengers, and 312 replies have been received. From the replies, two alerts were detected (Fig. 5).

2. Initiation of electronic community event-based surveillance system (eCBS)

The purpose of this system was to ensure a rapid detection, early warning, and prompt response to unusual events of potential threat to public health (i.e., SUDV), with emphasis on early implementation of basic control measures to reduce the risk of communicable disease spread in communities. An organized system for community health workers (CHWs) to detect local community events that may be dangerous and constitute a potential risk to public health and to quickly inform authorities was initiated to detect SUDV suspected cases as early as possible.

The eCBS system allows CHWs to notify any public health emergencies through short message services (SMSs) which are sent using ordinary mobile phones. When an SMS is sent, the community health worker supervisors at the health center level, district hospital level, and national level receive an alert which is immediately verified, and some action is taken.

At the end of December 2022, community health workers from SUDV high risk districts and other priority districts (Nyagatare, Gicumbi, Burera, Musanze, Rubavu, Nyabihu, Kicukiro, Gasabo, Bugesera, Gatsibo, Nyanza, Nyaruguru, Nyamagabe, Rwamagana, and Kirehe) were trained on the use of eCBS. Since the initiation of the eCBS system in Rwanda during the SUDV preparedness phase, around 378 signals of people presenting EVD likely symptoms and community deaths were observed. All these signals were notified through the system, and all the cases have been investigated. No Ebola case was identified.

#### Strengthening the Laboratory Capacity

Laboratory capacity is critical for the early detection and confirmation of SUDV cases. During the preparedness phase, laboratory capacity for SUDV testing in health facilities was strengthened through procuring and availing diagnostic tests where 33 kits that can test 3168 samples were procured, training of laboratory personnel and renovating satellite laboratories or testing hubs in provinces to boost the testing capacity of the National Reference Laboratory. Seven testing hubs were established including Ruhengeri RH, Rwamagana DH, Gisenyi DH, Kibuye RH, Gihundwe DH, and CHUB. The collected samples were immediately sent to a testing hub. The testing turnaround time was 8 h. From September 2022 to December 2022, a total of 250 samples had been tested countrywide for SUDV, and all tested negative.

### Capacity Building of Healthcare Providers on Case Management and Infection Prevention and Control (IPC)

#### Healthcare Providers Training

Training of healthcare providers on case management and infection prevention and control (IPC) was also a critical aspect of the SUDV preparedness phase. Healthcare providers including medical doctors, nurses, and paramedical staff from all health facilities and all private clinics from high-risk districts, as well as some selected private clinics in the city of Kigali, were trained and equipped with the knowledge and skills needed to effectively manage SUDV cases and prevent the spread of the disease.

**Fig. 6:**
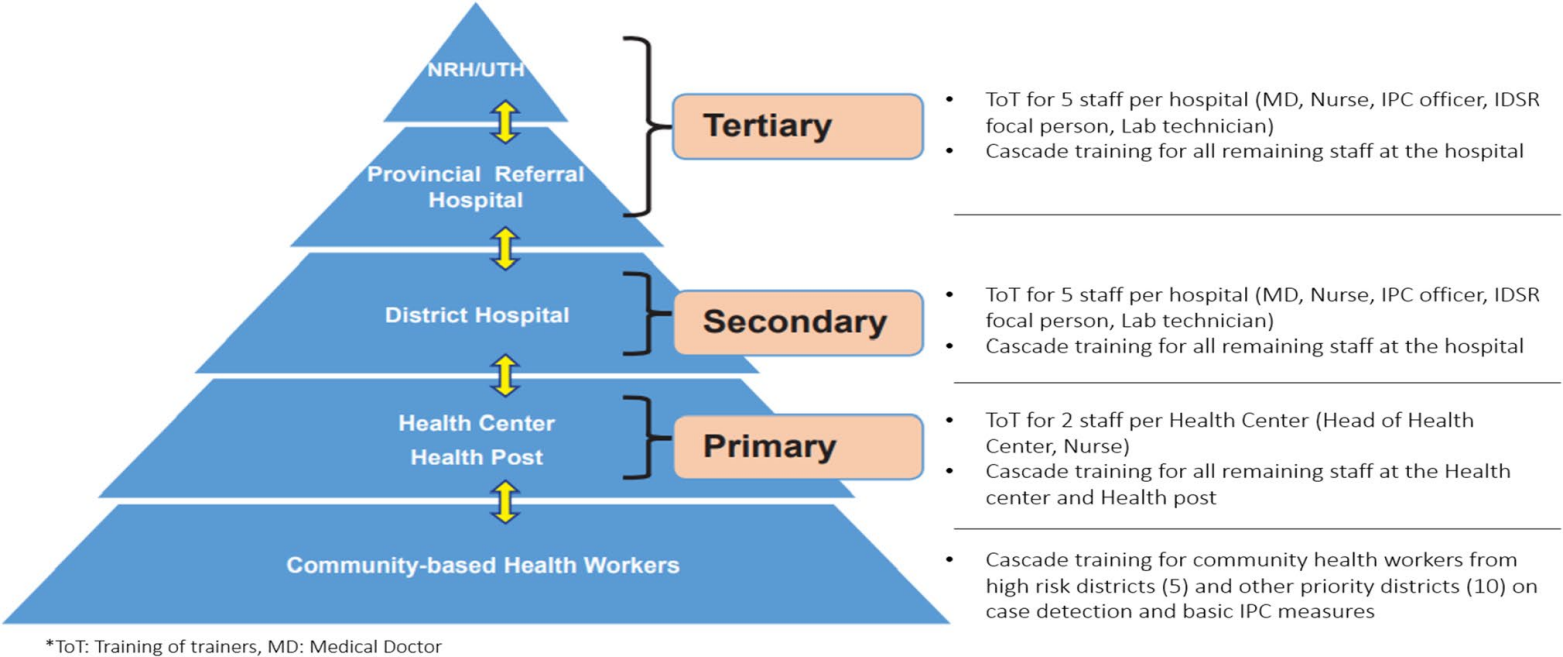
Levels of services provided within the public healthcare system

Healthcare providers were trained on the clinical features of SUDV, including its signs and symptoms, and how to diagnose and manage EVD cases. The training covered topics such as supportive care, hydration, nutrition, and pain management. They also received training on IPC measures to prevent the spread of EVD. The training covered topics such as hand hygiene, use of PPE, cleaning and disinfection of equipment and surfaces, and waste management. In addition, they were trained on how to provide psychological support to patients and their families during an SUDV outbreak. The summary on how healthcare providers from all health facilities were receiving training is described in Fig. 6.

#### Simulation Exercise

Simulation exercises were one of the crucial components of SUDV preparedness activities conducted among health facilities. These exercises involved creating scenarios that simulate an outbreak of SUVD and allow health workers to practice their skills and evaluate their preparedness to respond to an actual outbreak. These exercises were conducted among all 52 hospitals of Rwanda, and in some areas, exercises have been initiated at the level of health centers and referral processes.

Simulation exercises helped to identify gaps in preparedness plans, policies, procedures, and resources. They provided an opportunity for health workers to practice responding to SUDV outbreak and identify weaknesses in their response plans. These identified weaknesses were then addressed under the responsibility of the Director Generals of hospitals. Through simulation exercises, the team cohesion and collaboration among health workers was improved, and it was an opportunity for different pillars in the rapid response team to work together and practice their roles and responsibilities during an outbreak. Finally, it also improved communication and coordination in the case of an actual outbreak.

### Case Management, IPC and Contact Tracing

SUDV preparedness in Rwanda emphasized on training of healthcare providers, especially medical doctors and nurses, in robust SUDV case management. However, the country’s strategy for isolating all Ebola Virus Disease cases to prevent disease spread was to leverage on existing isolation rooms in hospitals as a primary part of this plan, in addition an Ebola Treatment Center located in Bugesera district was prepared to receive EVD cases. This facility, initially constructed during the COVID-19 pandemic, was dedicated to isolating patients during health emergencies, further enhancing Rwanda’s capacity to manage infectious diseases.

Infection prevention and control (IPC) was another critical aspect of Rwanda’s preparedness strategy. The country undertook extensive measures to provide healthcare providers with comprehensive IPC training. Alongside this training, materials and equipment necessary for effective IPC were supplied to all health facilities and community health workers, equipping them with the necessary tools to prevent SUDV transmission.

Rwanda planned also a rigorous policy of no home-based care or quarantine for SUDV. The country’s approach involved quarantining all SUDV contacts in designated sites. This comprehensive strategy was bolstered by prior mapping of quarantine sites across each district, ensuring readiness to enact quarantine measures swiftly when required.

### Risk Communication and Community Engagement

Effective risk communication and community engagement helped raise awareness and facilitate the adoption of preventive measures and safe behaviors among communities. During the SUDV preparedness phase, there was an engagement with key stakeholders, including government officials, community leaders, health workers, and civil society organizations. These stakeholders facilitated communication and engagement with communities and ensured that messages were tailored to local contexts by ensuring the messages provided were clear, concise, and accessible to all members of the community. The transmitted message included details about symptoms of SUDV, measures to prevent its spread, procedures for reporting individuals showing symptoms, and incidents of deaths within the community. Communication channels were diverse and tailored to the local context including community meetings, radio and television broadcasts, social media, and mobile messaging and posters.

Community engagement played a crucial role in enhancing the surveillance and prompt detection of EVD outbreaks. This involved raising awareness about SUDV and fostering community involvement in preparedness initiatives, such as practicing preventive measures and promptly reporting to nearby healthcare facilities when potential EVD symptoms presented. This was also a key component in strengthening surveillance in the community to identify potential SUDV cases early and promote effective community-based surveillance.

## Discussion and Reflections on the Lessons Learned

The Sudan Ebola Virus outbreak in Uganda was a significant public health event that required a coordinated response from the Ugandan government, international organizations, and other stakeholders. The response to the outbreak was swift, with the Ugandan Ministry of Health activating its emergency operations center (EOC) and working closely with the World Health Organization (WHO) and other partners to coordinate response efforts. The Ministry of Health established a national task force and regional Ebola coordination centers, which played a crucial role in detecting and responding to the outbreak [6]. The preparedness efforts in Rwanda, as one of the neighboring countries of Uganda, included enhancing the coordination mechanisms, strengthening surveillance and early detection, and increasing the capacity of healthcare providers on case management and IPC. The Rwanda Ministry of Health also implemented measures to prevent the spread of the virus, including screening travelers at border crossings and health facilities, and conducting public awareness campaigns to educate the public about the disease.

During the 2014–2016 Ebola Virus Disease outbreak in West Africa, coordination and leadership challenges hampered response efforts. The outbreak highlighted the need for effective leadership and coordination across sectors and among different stakeholders [10]. In contrast, during the 2018 Ebola Virus Disease outbreak in the DRC, effective coordination and leadership strategies were implemented. The response efforts in the DRC were led by the national government, which established a national Ebola response committee and a multi-sectoral coordination system. The DRC government also worked closely with international partners, including the World Health Organization (WHO) and the United Nations, to coordinate response efforts. These efforts were instrumental in containing the outbreak [11]. Similarly, in Uganda’s 2022 Ebola Virus Disease outbreak, effective leadership and coordination strategies were implemented [6]. Effective leadership and coordination strategies are critical in preparedness and response efforts to emerging infectious diseases. Lessons learnt from the Ebola Virus Disease outbreaks in different countries highlight the importance of effective coordination mechanisms. The country of Rwanda invested in building and maintaining robust health systems and effective coordination mechanisms during this phase of SUDV preparedness to ensure a coordinated and effective response to emerging infectious diseases.

The effectiveness of surveillance and early detection measures can vary depending on a range of factors, including the capacity of the health system, the resources available, and the local context. Liberia experienced a major outbreak of EVD in 2014–2016, which highlighted the importance of strengthening surveillance and early detection measures. Following the outbreak, Liberia implemented a range of measures to strengthen its surveillance system, including the establishment of a National Public Health Institute and the development of a National Health Security Strategy. These measures helped to improve the capacity of the health system to detect and respond to potential outbreaks of EVD [12]. The DRC has experienced multiple outbreaks of EVD, with the most recent outbreak occurring in 2021. To strengthen its surveillance and early detection measures, the DRC has implemented a range of measures, including the use of mobile laboratories for rapid diagnosis, the establishment of community-based surveillance systems, and the use of mobile phone technology to report suspected cases [13]. Sierra Leone also experienced a major outbreak of EVD in 2014–2016, which led to significant improvements in the country’s surveillance and early detection measures. This included the establishment of a National Ebola Response Center and the development of a national surveillance system for EVD [14]. Uganda has also experienced multiple outbreaks of EVD. To strengthen its surveillance and early detection measures, Uganda has implemented a range of measures, including the establishment of a national task force for EVD preparedness and response, the development of a national risk communication strategy, and the establishment of a surveillance system for EVD [6]. The outbreaks in other countries highlighted the critical role of public health infrastructure, including surveillance systems, laboratory capacity, and healthcare worker training, in detecting and responding to outbreaks. The country of Rwanda has a routine running disease surveillance system. By learning from experiences in different countries that have encountered EVD outbreaks, the country has strengthened the surveillance at point of entries and in health facilities. In addition, it established early warning systems, mainly in the community, to facilitate early identification of any event at the lowest level.

Training and strengthening the capacity of healthcare workers proved to be essential to prevent transmission and provide effective care for SUDV patients. During the 2014–2016 EVD outbreak, Sierra Leone implemented a range of measures to build the capacity of healthcare providers in case management and IPC. This included the establishment of training programs for healthcare workers on EVD case management and IPC, the deployment of international medical teams to provide training and support, and the provision of personal protective equipment (PPE) to healthcare workers [10]. To build the capacity of healthcare providers in case management and IPC, Uganda implemented a range of measures, including the development of national guidelines for EVD case management and IPC, the establishment of training programs for healthcare workers, and the provision of PPE to healthcare workers [6]. Guinea experienced a major EVD outbreak in 2014–2016, which led to significant efforts to build the capacity of healthcare providers in case management and IPC. This included the establishment of training programs for healthcare workers on EVD case management and IPC, the provision of PPE to healthcare workers, and the deployment of international medical teams to provide training and support [15]. To build the capacity of healthcare providers in case management and IPC, the DRC has also implemented a range of measures, including the establishment of training programs for healthcare workers, the provision of PPE to healthcare workers, and the deployment of international medical teams to provide training and support [11]. The country of Rwanda has implemented a range of measures during SUDV preparedness phase to improve the capacity of healthcare providers, including the establishment of training programs, the provision of PPE and conducting simulation exercises among health facilities. These measures have helped to improve the capacity of healthcare providers to manage SUDV cases and prevent transmission.

Engaging with communities helped to build trust, improve understanding of the disease, and increase compliance with public health measures. Sierra Leone implemented a range of measures to engage with communities and communicate information about EVD. This included the establishment of community engagement teams, the use of traditional and religious leaders to disseminate information, and the development of targeted messages for different audiences. Guinea experienced a major EVD outbreak in 2014–2016, which led to significant efforts to engage with communities and communicate information about EVD. This included the establishment of community engagement teams, the use of traditional and religious leaders to disseminate information, and the development of targeted messages for different audiences. To engage with communities and communicate information about EVD, the DRC has implemented a range of measures, including the establishment of community engagement teams, the use of community radio stations to disseminate information, and the development of targeted messages for different audiences. Similarly, in Uganda, to engage with communities and communicate information about EVD, they implemented a range of measures, including the establishment of community engagement teams, the use of traditional and religious leaders to disseminate information, and the development of targeted messages for different audiences. The country of Rwanda has also implemented a range of measures to engage with communities and communicate information about Ebola, including the engagement with key stakeholders to disseminate information and the development of targeted messages for different audiences through radios, TV, posters, and community meetings. These measures have helped to build trust and increase compliance with public health measures.

The SUDV preparedness phase also highlighted the importance of partners’ cooperation during outbreak preparedness. The country was able to work effectively with international partners and leverage their resources to better be able to prepare for the outbreak. In addition, the EVD outbreak underscored the importance of having a One Health approach to outbreak preparedness and response, as well as a need for surveillance systems that monitor both animal and human health. Rwanda was able to adopt a One Health approach and engage multiple sectors in outbreak preparedness and response to better detect and respond to outbreaks. A regular surveillance for EVD among fruit bats was initiated by Rwanda Development Board through the Veterinary Unit [16].

## Conclusion

The SUDV outbreak in Uganda has provided several lessons on preparedness and response to future outbreaks. These lessons are based on experiences and best practices observed during the SUDV preparedness phase in Rwanda. Strengthening healthcare systems and timely and effective surveillance systems, supported by laboratory capacity, are each essential to detect outbreaks early and contain them before they spread. It is also important to involve communities in outbreak preparedness efforts to improve understanding of the disease and increase compliance with public health measures. The adequate training and capacity building of healthcare providers is crucial to effectively manage EVD cases, prevent transmission, and protect healthcare workers. The effective coordination and collaboration between different stakeholders, including governments, non-governmental organizations, and international partners was also determined to be essential for a successful preparation to an outbreak. Finally, it was noted that it is important to maintain readiness for outbreaks even in the absence of one, to prevent future outbreaks and minimize their impact. Finally, our study concludes that proactive measures should be taken into account to sustain the preparedness for any potential outbreaks in the future, to reduce their impact.

## Data Availability

Not applicable.

## Abbreviations

CHW: Community health workers
COVID-19: Coronavirus disease 2019
DRC: Democratic Republic of Congo
eCBS: Electronic community event-based surveillance system
EVD: Ebola virus disease
IAEA: International atomic energy agency
IEC: Information education communication
IPC: Infection prevention and control
ITU: International telecommunication union
POE: Point of Entries
PPE: Personal protective equipment
SUDV: Sudan Ebola virus disease
WELTEL: Wireless emergency telemedicine
WHO: World health organization
